# Targeted sequencing library preparation by genomic DNA circularization

**DOI:** 10.1186/1472-6750-11-122

**Published:** 2011-12-14

**Authors:** Samuel Myllykangas, Georges Natsoulis, John M Bell, Hanlee P Ji

**Affiliations:** 1 Division of Oncology, Department of Medicine, Stanford University School of Medicine, Stanford, CA, 94305, USA; 2 Stanford Genome Technology Center, Stanford University, Palo Alto, CA, 94304, USA

## Abstract

**Background:**

For next generation DNA sequencing, we have developed a rapid and simple approach for preparing DNA libraries of targeted DNA content. Current protocols for preparing DNA for next-generation targeted sequencing are labor-intensive, require large amounts of starting material, and are prone to artifacts that result from necessary PCR amplification of sequencing libraries. Typically, sample preparation for targeted NGS is a two-step process where (1) the desired regions are selectively captured and (2) the ends of the DNA molecules are modified to render them compatible with any given NGS sequencing platform.

**Results:**

In this proof-of-concept study, we present an integrated approach that combines these two separate steps into one. Our method involves circularization of a specific genomic DNA molecule that directly incorporates the necessary components for conducting sequencing in a single assay and requires only one PCR amplification step. We also show that specific regions of the genome can be targeted and sequenced without any PCR amplification.

**Conclusion:**

We anticipate that these rapid targeted libraries will be useful for validation of variants and may have diagnostic application.

## Background

Next generation DNA sequencing (NGS) has revolutionized genetics by enabling one to routinely sequence genomes, either in their entirety or specific subsets. NGS technologies require complex sample preparation steps that involve addition of sequencer-specific DNA adapters to the genomic DNA fragments. Genomic DNA shearing, a series of molecular biology reactions (end-repair, A-tailing, adapter ligation and in most cases, PCR) and subsequent physical separation steps and purifications are typically performed to create the "sequencing library". For example, the Illumina Genome Analyzer utilizes partially complementary double-strand DNA adaptors that are used for PCR amplification and incorporation of common sequencing components to fragmented DNA [1]. Various groups have worked on streamlining the process and on reducing the amplification requirements for NGS libraries [2-4].

Targeted resequencing has proven useful for a large number of applications including validating variants from whole genome sequencing, studying disease-relevant gene subsets and diagnostic detection of clinically actionable variants. Along these lines, a variety of methods have been developed to enrich specific regions of the genome. These include hybrid selection, multiplex-PCR and targeted circularization approaches. Hybrid selection methods apply immobilized oligonucleotides on either microarrays [5-7] or beads [8] for the enrichment of genomic targets from a modified DNA sample. In multiplex-PCR [9], complex primer sets can be utilized to selectively amplify targeted regions prior to modifying DNA for the sequencer. Recently, microdroplet technology [10] has been utilized to effectively parallelize individual PCR reactions. Molecular inversion probes capture inversion probes use polymerase extension across the target and ligation to circularize, thus enabling highly multiplexed targeted resequencing [11,12]. Targeted genomic circularization directly captures one strand of the genomic DNA target by converting the target fragment DNA into a circle using in-solution capture oligonucleotides [13]. More recently, Oligonucleotide-Selective Sequencing which is a target-specific hybridization and extension approach for capturing genomic target sequence has been developed [14]. Nearly all of these methods require the added step of ligating sequencing adapters either pre- or post-enrichment.

**Figure 1:**
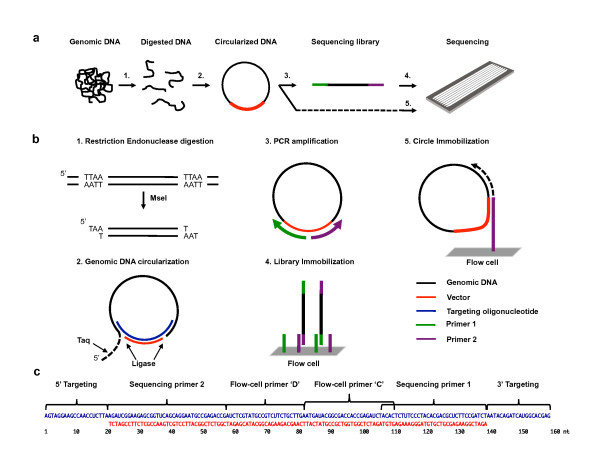
**Targeted sequencing library preparation method**. **(a) **Overview of the assay. **(b) **Specific preparation steps: (1) genomic DNA is digested using *Mse*I restriction endonuclease. (2) Then, genomic DNA fragments are circularized using thermostable DNA ligase and Taq DNA polymerase for 5' editing. Pool of oligonucleotides targeting 5' and 3' ends of the DNA fragments and vector oligonucleotide are used for targeted DNA capture. (3) After circularization, regular Illumina sequencing library can be prepared by PCR. (4) PCR amplified library fragments are similar to regular Illumina library constructs and anneal to immobilized primers on the flow cell. (5) Additionally, circular constructs can be directly sequenced as the adapted genomic DNA circles incorporate all DNA components required for library immobilization and sequencing. **(c) **Molecular structures of vector oligonucleotide and capture oligonucleotides.

Because the target enrichment and sequencing library preparation occur in succession, targeted resequencing methods tend to be laborious, time-consuming, prone to experimental errors and difficult to automate. In addition, many of these approaches have complex protocols which require large amount of starting genomic DNA. Other issues include being susceptible to coverage biases secondary to amplification and molecular artifacts. Herein, we demonstrate a method of integrating both NGS library preparation and capture of specific genomic targets. We have developed an approach in which circularization of a specific genomic DNA molecule directly incorporates Illumina sequencing library components (Figure 1). Described simply, a target genomic DNA fragment is circularized. All of the sequencing library components are included in the circularization step. Once a specific target circle forms, all of the components of the Illumina sequencing library are generated in single PCR amplification step that provides a targeted library that is ready for sequencing. This single-step method reduces the complexity associated with targeted resequencing. We also demonstrate the technical feasibility of amplification-free targeted resequencing.

## Results and Discussion

### Overview of preparing targeted sequencing library by genomic circularization

To test this method of targeted library creation we initially used targeted genomic circularization as has been recently described by Natsoulis et al. [13] although our library creation method is applicable to other circularization approaches such as molecular inversion probes. Targeted genomic circularization requires a restriction endonuclease digest of nanogram amounts of genomic DNA followed by incubation with a mixture of two types of oligonucleotides. These are: i) a pool of capture oligonucleotides which are specific for a targeted genomic region and ii) a universal vector oligonucleotide [13]. Each capture oligonucleotide is an 80-mer and has two target-specific complementary end-sequences (20 nucleotides each) at the flanks. The target-specific complementary flank sequences are referred to as capture arms. Between the capture arms is a general sequence motif (40 nucleotides) that is found in every capture oligonucleotide. The universal vector (40 nucleotides) has the complementary sequence to the general sequence motif found in each individual capture oligonucleotide.

Once the genomic DNA has been fragmented with a specific restriction endonuclease such as *Mse*I, the capture oligonucleotide pools and genomic DNA are incubated together in a single reaction volume (Figure 1). For each oligonucleotide, the complementary flank sequences, referred to as capture arms, hybridize to the target genomic DNA molecule and mediate the selective circularization of single stranded target DNA (Figure 1a and 1b). The capture oligonucleotide acts as a "splint" that anneals and thus bridges the two ends of the fragment and this leads to a partial circularization of the fragment. The 3' end of the targeted genomic DNA fragment is required to align and hybridize perfectly with the capture and vector oligonucleotides. However, the 5' end of the fragment may contain an overhang which is subsequently cleaved by Taq DNA polymerase during the circularization reaction [13]. This relaxes the criteria for designing capture oligonucleotides, since only one of the two ends requires a specific restriction endonuclease site. We provide detailed description including DNA sequences and corresponding molecular biology of targeted sequencing library preparation assay in Additional File 1.

To complete the circle, the universal vector oligonucleotide anneals to the general sequence motif in every targeting oligonucleotide and thus is guided to the ends of the circularized genomic DNA target. The universal vector is subsequently ligated to the two ends of the target DNA fragment, incorporated into the genomic DNA sequence and thus completing the target-specific circle. Once the circle is complete, universal PCR primers can be used to amplify out the intervening target genomic DNA fragment, creating a pool of linear amplicons that can be sequenced. We engineered the components of the Illumina sequencing adapters into the universal vector sequence such that a single PCR amplification of the target-specific circles leads to a ready-to-run sequencing library (Figure 1b, c). A target-specific circle is required for the primers to amplify the fragment.

### Design of the target-specific capture oligonucleotides

For this proof-of-concept study, we selected 107 capture oligonucleotides to capture exon regions of 10 cancer-related genes from genomic DNA digested with *Mse*I (Additional File 2). The majority of these capture oligonucleotide sequences were selected from a general web-based resource called the Human OligoExome http://oligoexome.stanford.edu[13]. This public resource database contains predesigned capture oligonucleotide sequences for nearly all human exons as annotated by Consensus Coding Sequencing Project (CCDS) [15]. For this database resource, we designed capture oligonucleotides with substantial redundancy to increase the likelihood that at least one oligonucleotide would capture the target. Four restriction enzymes provided enough sites to adequately design capture oligonucleotides which cover over 98% of regions of interest (ROI) bases over all CCDS exons. Also a variety of different fragment sizes can be captured. Recently, we released an expanded version of this capture oligonucleotide resource which covers the majority of the human genome http://oligogenome.stanford.edu[16].

**Figure 2:**
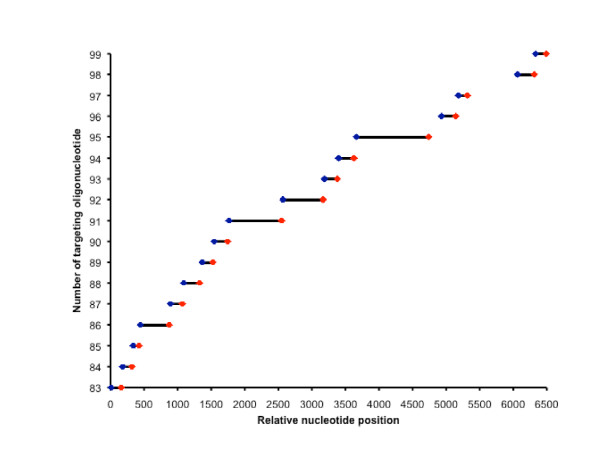
**Seventeen targeting oligonucleotides (numbers 83-99) were designed to tile across exon 15 of the *APC *gene**. 5' target sites are marked blue and 3' target sites are marked red. Intermediate circularized genomic DNA is marked using black lines.

As a demonstration of the capacity of our assay's capture, we included a subset of capture oligonucleotides that tiled across a large 6.5 Kb region in exon 15 of the *APC *gene (Figure 2). By design, our assay mediates end-sequencing of the targeted fragments and Figure 2 shows how capture regions can tile across uninterrupted regions in the genome such as exon 15 of *APC*. The capture oligonucleotides can be designed so that extended genomic targets such as large exons can be tiled across and thus sequenced in its entirety by NGS.

### Assessment of hybridization temperatures for circularization-library preparation

To optimize the performance of the 107 capture oligonucleotide assay, we tested three different hybridization temperatures at 60, 55 and 50°C which are listed in Table 1 as Experiments 1, 2 and 3 respectively. From our previous study, hybridization temperature was one of the most critical factors for targeting [13]. Given the inclusion of the sequencing library components into the capture oligonucleotides, our goal was to identify an optimal temperature range for the assay. Using these different temperatures, we completed the following steps to create the targeted sequencing libraries. First, we digested normal diploid genomic DNA using the *Mse*I restriction endonuclease (Figure 1a and 1b). Second, we used a pool of capture oligonucleotides as target-specific splints and circularized the target genomic DNA fragments by double-ended ligation to a common vector oligonucleotide. We carried out 15 circularization cycles using a thermostable ligase. Third, we amplified linear sequencing library amplicons from the circles using the universal primers located in the vector sequence (Figure 1c).

**Table 1:** Results from targeted sequencing libraries

Experiment	1	2	3	4	5
Hybridization temperature (°C)	60	55	50	55	60
Number of PCR cycles	25	25	25	Direct	25
Sequencing read length (mate pair versus single)	42	42	42	42	42 by 42
					
Total reads	12,542,683	15,605,713	12,435,664	1,232,093	33,750,858
Mapped reads **^a^**	8,576,700	13,415,111	7,381,662	11,726	31,325,015
Captured on-target fragment-end reads **^c^**	7,560,090	11,105,527	6,330,012	8,488	30,994,237
Captured off-target reads (as % of mapped)	1,016,610 (12%)	2,309,584 (17%)	1,051,650 (14%)	3,238 (28%)	660,937 (2%)
					
On-target fragment-end sequences (bases) **^c^**	4,410	4,410	4,410	4,410	8,778
Captured on-target fragment-ends with 1× (bases) **^c, d^** (as % of on-target region-of-interest)	3,145 (71%)	3,340 (76%)	3,044 (69%)	2,809 (64%)	6,628 (75%)
Captured on-target fragment-ends with 30× (bases)**^b, c^** (as % of on-target region-of-interest)	2,932 (66%)	3,128 (71%)	2,961 (67%)	2,160 (49%)	6,460 (73%)
Average on-target fragment-end fold-coverage	72,001	105,767	60,286	81	149,100

**^a ^**ELAND alignment. **^b ^**Sequencing fold-coverage > 30. **^c ^**Compilation of 42-base end-sequences from circularized targets. **^d ^**Sequencing fold-coverage > 1.

**Figure 3:**
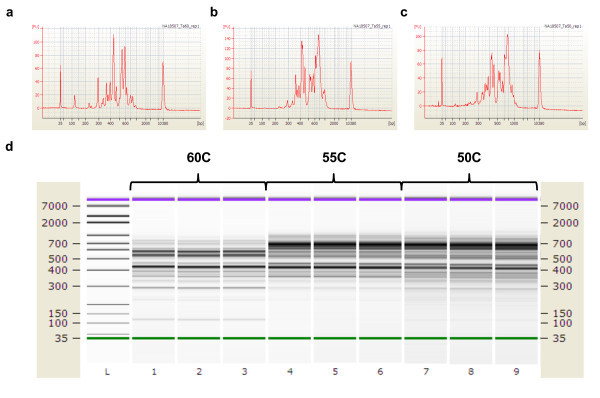
**Bioanalyzer analysis of the size distribution of sequencing libraries**. Targeted sequencing libraries were prepared by circularization and the size distributions are shown for the following three conditions **(a) **60C, **(b) **55C, and **(c) **50C. **(d) **The electrograms for replicates clearly indicate that the size of the targeted sequencing libraries increases with lower hybridization temperatures.

Afterwards, we used standard size-filtration column purification for sequencing libraries immediately before starting the sequencing process. This entire targeted library preparation did not require any extraction or gel purification and was completed in a single day. We used an Agilent Bioanalyzer to analyze the size distribution of the sequencing libraries. Our analysis revealed that different hybridization temperatures during circularization affect the fragment size pattern of the captured circles (Figure 3a and 3b). The electropherograms for the experimental replicates clearly indicate that the size of the targeted sequencing libraries increases with lower hybridization temperatures.

To determine the general performance of the temperature hybridization experiment, we used single read sequences as "tags" to assess the fragment-ends of the captured DNA target. This involves counting the target fragment-end sequence(s) for quantitatively assessing the efficiency of capture. Using the same molar concentration, each experimental capture library was sequenced on a single Illumina GAIIx lane with single 42 base reads. We subsequently aligned the data to the human genome reference for assessment of target specificity. The sequence data characteristics are listed in Table 1.

Overall, sequence quality from the targeted libraries (Experiments 1 through 3) was high with as many as 93% of the total reads mapping to the human genome. Average sequencing fold coverage for targeted regions was in the range of tens of thousands for these targeted sequencing libraries regardless of the hybridization temperature (Table 1). More on-target sequences with overall higher on-target coverage were demonstrated at a 60 and 55°C hybridization temperature compared to 50°C (Table 1). Specifically, hybridization at 60 and 55°C resulted in higher capture coverage at 1× or greater of the specific targets (71 and 76%, respectively) compared to 50°C (69%).

**Figure 4:**
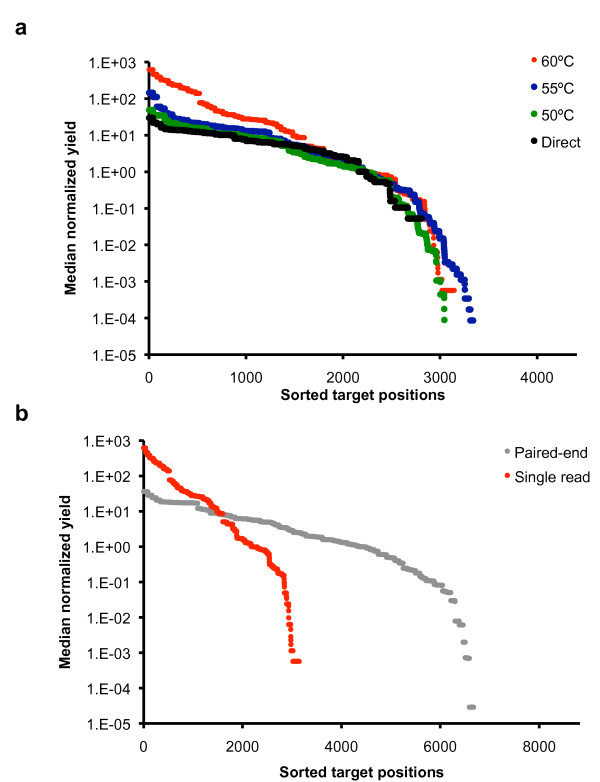
**Uniformity of capture coverage**. Uniformity of the coverage in **(a) **single-end sequencing libraries (Experiments 1 - 4) and in **(b) **paired-end sequencing library (Experiment 5) and single-read sequencing library (Experiment 1) is presented. In the figures, median normalized sequencing fold-coverage (y-axis) is presented for each targeted position (y-axis). Targeted region in (a) was 4,410 bases and targeted region in (b) was 8,904 bases.

To assess the overall uniformity of the capture over the different hybridization temperatures, we sorted the yield of the capture oligonucleotides by increasing fold coverage per base and plotted the results (Figure 4a). Our data shows that lower hybridization temperature during circularization results in more even coverage between different capture oligonucleotides (Figure 4a). For example, at 60°C, there is clearly a wider range of variation compared to 50 or 55°C.

Analysis of these libraries revealed that different hybridization temperatures affect the efficiency of circularization and the sizes of fragments that can be captured. This suggests that properties of the 20-mer targeting arms define an optimal annealing temperature for each oligonucleotide. Overall, this analysis identified the hybridization temperature range to create targeted sequencing libraries using our single step method. Our initial proof-of-concept demonstration used 107 capture oligonucleotides to generate single step sequencing libraries. The complexity of the assay and the size of the target region can be increased by using multiple restriction endonucleases in the genomic digestion step and by adding more capture oligonucleotides. We have previously shown that thousands of oligonucleotides can be used in targeted genomic circularization [13], suggesting that with this direct library method that one Mb of DNA can be targeted, captured and rapidly converted to a sequencing library.

In terms of extending the fragment-end sequencing to cover the entire amplicon, with the Illumina HiSeq and MiSeq system it is possible to sequence 300 base target regions using 150 by 150 paired-end reads. As we have previously demonstrated, we can use targeted genomic circularization for targeted resequencing on the Roche 454 system [17]. In this earlier work, we leveraged the longer reads of the 454 system to sequence the amplicons. Generally, since that earlier effort, we have dramatically improved the performance of the capture assay with greater capture coverage up to 1 Mb, decreased template requirement of under 100 ngs and methods to reduce variance of capture [13].

### Direct sequencing of PCR amplification-free circular targeting libraries

Using the 107 capture oligonucleotide assay, we determined if one could eliminate the PCR amplification of the targeted circles. Essentially, this test relied on the same steps for targeted library preparation but excluding the final PCR amplification step (Figure 1b). This unamplified library was added directly to the Illumina flow cell. This experiment is listed as Experiment 4 in Table 1 and used the same source and amount of genomic DNA source as Experiments 1 through 3.

We conducted the analysis of this amplification-free targeted sequencing library with a single sequencing lane using a 42 base single read. As described previously, after alignment, we assessed the targeted library by counting the sequences from the captured fragment ends. The direct sequencing of this amplification-free targeted library produced a total of 8,488 sequence reads. The yield was low given the relative small number of target genomic DNA molecules available for sequencing. Average sequencing fold-coverage for the targets was over 80×. Similarly, 64% of target regions-of-interest was covered by at least one read and 49% of the target regions-of-interest had greater than 30×. Direct sequencing of the circularized DNA without PCR amplification yielded the most off-target sequences (28%) because a high proportion of reads derived from adapter sequences.

We assessed the uniformity of coverage of the amplification-free library and determined that it had relatively even coverage (Figure 4a). This suggests that PCR amplification of the targeted sequencing libraries contributes to skewing the number of captured targets as seen in Experiments 1 through 3 (Figure 4a). Compared to the amplified libraries (Experiments 1 through 3), the on-target yield was less due to the small number of molecular targets in the human genomic DNA sample.

We clearly demonstrate that it is possible to directly sequence circularized target DNA without PCR amplification. The major limitation with amplification-free capture is the small number of target DNA fragments, which were captured as quantified by on-target sequence reads. By targeting 107 loci we captured and sequenced ~8,500 circles (Table 1). Increasing the number of oligonucleotides will clearly improve this capture coverage and given that we previously demonstrated that thousands of targets can be circularized, the number of sequences can be increased by at least 10-fold with an increase in oligonucleotides [13]. However, one must increase the number of circles by three orders of magnitude to utilize fully the capacity of even the lower-end NGS systems. For example, the Illumina MiSeq Personal Sequencing System currently analyzes 5-7 million fragments. Therefore, the advantages of amplification-free sequencing become evident when assays using over 100,000 oligonucleotides are available. In addition to incorporating a larger number of capture oligonucleotides, improvements in the purification of targeted circle purification will certainly improve the performance of direct PCR-free sequencing. For example, we observed a significant fraction of the total sequences (Table 1) originated from sequencing adapters. New purification methods and optimization of the concentration of targeting oligonucleotides in the circularization reaction should be examined for future improvements.

### Assessment of the overall capture coverage with mate pairs

With the 107 capture oligonucleotide assay, we use paired-end sequencing to assess the assay performance and our tiling approach for exon 15 of *APC*. The results are noted as Experiment 5 (Tables 1 and 2). To determine the capture coverage over exon 15 of *APC*, we counted the number of target fragment-end reads that were located in exon 15 of *APC *(Table 2). These data demonstrate that targeted sequence was derived from 16 out of 17 genomic targets. Two capture oligonucleotide failed to capture their targets. Capture oligonucleotide 95 failure was the result of a large target genomic fragment which was 1.1 Kb in length (Additional File 2). Previously, we had determined that an optimal size was 0.8 Kb or smaller [13]. The second failure from capture oligonucleotide 97 may have been the result of a chimeric product that likely occurred during the amplification.

**Table 2:** Sequence counts for oligonucleotides capturing *APC *exon 15

Capture oligonucleotide number	Target fragment-end sequences (amplification)	Target fragment-end sequences (direct)
83	281,845	265
84	77,827	231
85	170,663	263
86	587,011	152
87	16,263	380
88	301,843	200
89	366,455	564
90	63,568	219
91	3,664	95
92	213,413	121
93	629,858	261
94	831,320	272
96	5,010	19
98	236,224	187
99	973,662	214

For Experiment 5, overall on-target average fold coverage of fragment-ends was 149,100×. This was higher than Experiments 1-3 because we used paired-end reads to determine fragment-end composition from the captured targets. Subsequently, we analyzed the regional coverage of the targets. We calculated that 75% of the target region was captured at least once and 73% of the targeted bases were captured with fold-coverage above 30× (Table 1). The difference in coverage between amplicon and single molecule sequencing reflects the overall lower sequencing depth of the direct circular library. Coverage across exon 15 of *APC *was complete with the exception of two target tiles.

With paired-end sequencing of a PCR amplified library (Experiment 5), we achieved high on-target specificity, as demonstrated by the fact that only 2% of the mapped reads were outside of the targeted regions (Table 1). Experiments 1-3 which used single reads showed slightly higher off-target rate (12 -17%) than paired-end sequencing. We also addressed the uniformity of the coverage from paired-end data by binning the paired-end reads for the individual capture oligonucleotides (Figure 4b). According to our results, paired-end sequencing improved the uniformity of the coverage when compared to single read experiment in similar capture conditions. These results suggest that paired-end sequencing provides a benefit in terms of increasing the accuracy of mapping.

### Evaluation of size in capture yield

**Figure 5:**
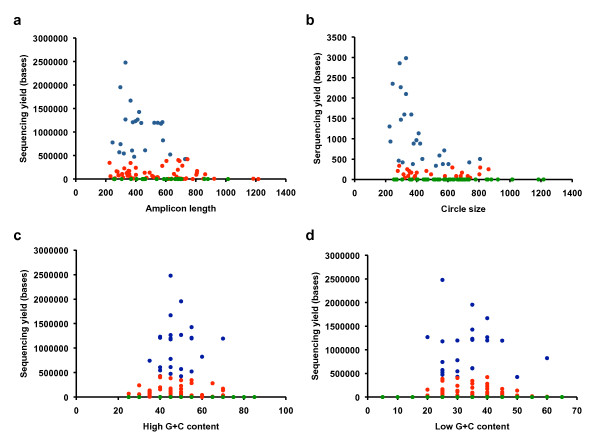
**Analysis of capture parameters and performance**. Relation between sequence read yield and **(a) **amplicon length in paired-end sequencing (Experiment 1), **(b) **circle size in direct amplification-free sequencing (Experiment 5), **(c) **high (G+C) content, and **(d) **low (G+C) content. Blue dots represent well performing oligos, red dots represent moderate performing oligonucleotides and green dots represent failed oligonucleotides.

Gaps in the coverage of the targets and variation of capture uniformity are directly associated with inefficiencies in the performance of specific capture oligonucleotides. We identified two reasons for failure: 1) target circularization fails due to unfavorable properties of the targeting sites, and/or 2) size of the captured template is unsuitable for sequencing. To investigate the capture properties of the assay we classified each capture oligonucleotide based on its specific sequence yield into one of three categories based on on-target sequence. A subset of the oligonucleotides (n = 25) captured no target sequence and they were classified as failures. Twenty-five of the oligonucleotides that captured the most sequences were considered to be high performing. Fifty-seven capture oligonucleotides produced moderate yields between the extremes of no capture or high capture and were considered to be of moderate performance. As determined by the target length, sizes of the captured circles (Additional File 2) were correlated to sequence yields (Figure 5). Amplicons between 200 and 600 bases perform robustly, while amplicons larger than 600 bases fail or result in low capture yields (Figure 5a). There are at least three factors that may combine to produce low yields in the larger amplicons: (1) inability of a capture oligonucleotide to create large circles, (2) a PCR-induced bias against larger circles at the amplification step, (3) reduced efficiency of cluster formation on the flow cell. We performed a similar analysis using data from the directly sequenced circular library (Experiment 5) and observed that similar correlation patterns between circle size and yield existed without PCR amplification (Figure 5b). This suggests that circularization and cluster formation contribute to the target size bias and not PCR amplification.

With our capture method, the entire sequence read is derived from genomic DNA and does not cross into synthetic sequence. Since the first 20 bases of the sequencing reads are complementary to the target specific sites, individual capture oligonucleotide species can be directly linked with sequencing data. Using the target specific sequence as a molecular barcode allows highly specific analysis of the performance of capture oligonucleotides. In addition, it is possible to incorporate index sequences directly into capture oligonucleotides and vectors or, alternatively, library amplification primers to enable sample multiplexing [13,18].

### Evaluation of G+C content in capture yield

We evaluated specific properties of the capture oligonucleotides, such as the guanine and cytosine (G+C) content of target specific 20-mer. We found that G+C content of the target specific sites (Figure 5c and 5d) was associated with sequence yields or total failure of the oligonucleotides. The G+C content of the targeting arms varied between 5 - 85%. The differences in targeting arm base composition influences the optimal annealing temperature varies among individual capture oligonucleotides in the pool. When circularization/hybridization experiments were carried out in 60°C, the optimal G+C content was observed in a range of 30 to 60%. At the optimal temperature for annealing at 55°C, a similar G+C content is also important for capture performance. We hypothesize that oligonucleotides with low G+C content do not properly anneal to targets during circularization. Conversely, high G+C content represses the denaturing of DNA during heat incubation.

We have demonstrated that simple optimization of the oligonucleotide design would improve the capture yields. For instance, the size of the circles should be restricted to 200-600 bases and G+C content of the 20-mer targeting sites should be limited to a range 30-60% for more uniform coverage. Targeted sequencing library preparation method is an integrated solution and oligonucleotide properties that affect both capture and sequencing should be taken into consideration in assay design.

## Conclusions

In this proof-of-concept study, we outline a novel strategy to prepare NGS libraries of targeted DNA content with a single circularization step and demonstrated the feasibility in this proof-of-concept study. Instead of amplifying the circles using a pair of universal primers and ligating Illumina adapters to the amplified material, we include all of the Illumina adapter sequences in the oligonucleotides that mediate the circularization. Targeted genomic circles can be generated and converted to a sequencing library using PCR amplification with standard Illumina primers. Integrating library preparation and target enrichment, our assay effectively captures targeted genomic regions with good coverage and high specificity. Furthermore, by streamlining the targeted resequencing process, the preparation time can be reduced to a single day.

For this proof-of-concept study, we sequenced the target fragment-ends for quantitative analysis of a given target. However, with steady improvements in sequencing technology, longer reads are becoming available from platforms such as the Illumina HiSeq and MiSeq system or the Roche 454 system. Therefore, it is possible to sequence over the entirety of a given target region. Given our ability to design capture oligonucleotides which are under 300 to 400 bases, this makes rapid targeted resequencing library preparation practical for covering exon targets in their entirety with single or mate pair reads. We are pursuing this strategy for future applications.

Although we demonstrated the feasibility of the method using the Illumina NGS system, our approach is generally applicable for generating sequencing libraries for different sequencing platforms. For example, the 454 (Roche) and the SOLiD (Applied Biosystems) platforms rely on preparing recombinant sequencing libraries that have specific adaptor sequences at 3' and 5' ends, and the PacBio RS system utilizes circular DNA as a template for sequencing. This suggests that the targeted circularization assay presented here might be applicable for variety of NGS systems.

In summary, targeted resequencing applications are expected to provide the foundation for clinical genomics and high-throughput genetic diagnostics. With our OligoExome [13] and OligoGenome resource [16], one can rapidly design and implement high-throughput targeted analysis and validation of disease-related variants in the human genome.

## Methods

### Oligonucleotides

The capture oligonucleotide sequences were extracted from the Human OligoExome Resource http://oligoexome.stanford.edu[13]. This site provides access to a database containing *in silico *capture oligonucleotide sequences covering 17,049 genes listed in the Consensus Coding Sequence Project (CCDS build 20080902 for hg 18) [15]. One can use this website resource to create customized capture assays. Overall, the Human OligoExome has a total of 784,783 capture oligonucleotides specific for four restriction enzymes including *Mse*I. Targeting arms were positioned in SNP-free regions as defined by a lack of overlap with dbSNP129. To improve capture performance, four different quality control factors were reviewed for each oligonucleotide which included the presence of sequences repeated over the human genome, paralogs, matches to consensus repeats and Alu sequences. Excluding the flagged oligonucleotides for all four enzymes provided an average coverage of 94.2% for any given CCDS annotated gene.

The *in silico *capture oligonucleotide sequences were modified to include the original two hybridization regions (20 nucleotides each) in the ends of the oligonucleotide and the addition of sequence components that correspond to forward (58 nucleotides) and reverse (61 nucleotides) Illumina paired-end adapters. The size of each capture oligonucleotide was 158 bp. At least one of the targeting arms coincides with the last 20 bp of an *Mse*I restriction fragment. When only one of the targeting arms is adjacent to a restriction site, the other end of the captured DNA strand forms a 5' phosphate extension, which is degraded during the circularization reaction by the 5'-exonuclease activity of Taq DNA Polymerase [19], thereby allowing Ampligase to form a single stranded circle.

A total of 107 capture were synthesized (Additional File 2). The 5' and 3' ends of the capture oliogonucleotides were blocked and did not contain phosphate or hydroxyl groups and ten Thymines were substituted with Uracils to facilitate fragmentation and purification of the splint oligonucleotides after circularization. In addition, the 119 nucleotide vector oligonucleotide was synthesized (Additional File 1). Vector oligonucleotide is complementary to the common portion representing the inverted Illumina adapters in the middle of the capture oligonucleotides. All oligonucleotides were synthesized at the Stanford Genome Technology Center Stanford, CA) and purified using Qiagen spin columns.

### Targeted genomic circularization

Genomic DNA obtained from NA18507 (Coriell Institute) was used for demonstration of targeted circularization based sequencing library preparation. We digested 1 μg of genomic DNA from NA18507 with *Mse*I restriction endonuclease (NEB) for 3 hours in 37°C, followed by a heat inactivation of the enzyme for 20 min in 65°C. *Mse*I digested genomic DNA was circularized in the presence of a pool of 107 genomic circularization oligonucletides (50 pM/oligo) and vector oligonucleotide (10 nM). Circularization experiments were carried out using Ampligase thermostable ligase (Epicentre) and Taq DNA polymerase (Invitrogen) was used for 5' flap processing. After heat shock denaturation of the sample in 95°C for 5 min, 15 circularization cycles (denature in 95°C for 2 min, hybridize in 60°C for 45 min and flap processing in 72°C for 15 minutes) were performed.

### Purification of captured genomic circles

Circles were purified by degradation of the genomic DNA fragments and excess linear oligonucleotides using a mixture of Exonuclease I and III enzymes (NEB) and incubating the reaction in 37°C for 30 min, followed by heat inactivation of the enzymes (80°C, 20 min). Samples were further digested using Uracil-Excision enzyme (Epicentre) to fragment the capture oligonucleotides. Size fractions corresponding to 300-1200 bases were extracted from circularized DNA preparations using Gel Extraction purification (Epicentre). Purified circles were eluted to a total volume of 30 μl.

### Preparation of the amplification libraries

10 μl of the purified circles were amplified using Phusion Hot Start DNA polymerase (Finnzymes, Finland) and general Illumina paired-end library preparation primers. We used the following PCR program: 25 cycles (98C, 10 s; 65C, 30 s; 72C, 15 s) followed by an extension step (72C, 5 min) were run. Amplified products (300 bp-1200 bp) were purified using a Fermentas Gel Extraction kit.

### Sequencing

10 pM of PCR amplified library and 1.5 pM of circularized DNA were sequenced using Illumina Genome Analyzer IIx. Circular library obtained from 1 μg of starting material was introduced to the sequencing experiment. After sample dilution using hybridization buffer, 20% of the prepared sample (representing 200 ng of starting material) was hybridized in the flow cell. Using the standard Illumina protocol, paired-end sequencing of 42 bases was performed using Illumina Genome Analyzer IIx.

### Data analysis

We used the ELAND program to align sequence reads to the human genome version hg18. We defined the target regions as the ranges from each target specific site to 41 bases upstream or downstream of it (depending on the orientation of the selector). The interval of 41 bases was selected because the read length in these experiments was 42. In a paired-end experiment the target region contained both ends of the circularized fragments, while single-read sequencing targeted only 3' ends of the circularized fragments. To assess the specificity of the capture we compared the numbers of sequence reads mapping inside and outside the target region. To illustrate the uniformity of the assay, we counted the reads that aligned perfectly with the specific capture sequences. Read counts were then sorted and normalized using the median sequence yield value from each experiment. The genomic distance between the target specific sites indicates the circle size. In addition, guanine and cytosine proportions within the target sites were determined. One capture oligonucleotide contains two target specific sites and each site was analyzed separately. To analyze the annealing properties during circularization-hybridization reaction, we classified target specific sites within a single capture oligonucleotide as high or low G+C. We plotted circle sizes and G+C proportions with the sequence yields for each oligonucleotide.

## List of abbreviations

NGS: next-generation sequencing; G+C: guanine and cytosine; GAIIx: Genome Analyzer IIx

## Authors' contributions

SM conceived the methods, participated in designing the study, performed molecular analyses, analyzed and interpreted the data. JMB performed sequence analysis. GN designed the target specific sites of the capture oligonucleotides. HPJ participated in methods development, designing the study and coordinated the overall study. All authors drafted, revised, read and approved the final manuscript.

## Supplementary Material

Additional File 1 **Molecular steps in targeted circular sequencing libraries**. This file contains a detailed DNA sequences and molecular biology descriptions of the targeted library assay. Click here for file

Additional File 2 **Oligonucleotide sequences**. This file includes the sequences and molecular properties of the capture oligonucleotides. Click here for file
