# Supplementary material for: Targeted sequencing library preparation by genomic DNA circularization
Source: BMC Biotechnol. 2011 Dec 14;11:122. doi: 10.1186/1472-6750-11-122 (PMC3280942; doi:10.1186/1472-6750-11-122)
Supplement: Additional File 1 — Molecular steps in targeted circular sequencing libraries. This file contains a detailed DNA sequences and molecular biology descriptions of the targeted library assay. [file 1472-6750-11-122-S1.PDF]

TARGETED SEQUENCING LIBRARY PREPARATION BY GENOMIC DNA CIRCULARIZATION

In the following, we are describing the method using MseI restriction enzyme digestion and paired-end sequencing. The process includes:
1. Genomic circularization
2. Cluster generation
3. Sequencing

1. GENOMIC CIRCULARIZATION

1.1. Genomic DNA

Genomic DNA contains 4-base restriction sites, TTAA, for MseI.

```
5' -  NNNNNNNNTTAANNNNNNNNNNNNNTTAANNNNNNNNN  - 3'
3' -  nnnnnnnnAATTnnnnnnnnnnnnnAATTnnnnnnnnn  - 5'
```

1.2. RE digest fragment by MseI

After restriction digest, genomic DNA fragments contain AT overhangs.

```
5' -           TAANNNNNNNNNNNNT             - 3'
3' -             TnnnnnnnnnnnnAAT           - 5'
```

1.3. Circularization

1.3.1 Hybridization of adapted genomic DNA and vector and targeting oligonucleotides

After MseI digestion genomic DNA fragments are formed. These fragments are targeted. For example, Minus-strand of fragment below is targeted using Oligo 1 in our assay.

```
Plus:   5' - TAAATACAGATCATGGCACGAGGCCGCCTCTCCATCCCTCCAAGCTATCGTCCAGCGCAGTCCACCGCCGCCTCAGGCCGTGCCGCTGGCCGAGTAGGAGAACTGGGGGAAGTGGGGCCTGCGCTCGCTGTCCACACACTCCATGCTGTCATCTGTGGGTGTAGACAGCTCAGACCCCGGTGCCCCACCTCCCTGCCACCTCCACCCACCCACAGCTCCAGTAGGAAGCCAACCTCT   - 3'
Minus:  3' -   TTATGTCTAGTACCGTGCTCCGGCGGAGAGGTAGGGAGGTTCGATAGCAGGTCGCGTCAGGTGGCGGCGGAGTCCGGCACGGCGACCGGCTCATCCTCTTGACCCCCTTCACCCCGGACGCGAGCGACAGGTGTGTGAGGTACGACAGTAGACACCCACATCTGTCGAGTCTGGGGCCACGGGGTGGAGGGACGGTGGAGGTGGGTGGGTGTCGAGGTCATCCTTCGGTTGGAGAAT - 5'
```

Genomic circularization oligonucleotide:

5' - AGTAGGAAGCCAACCTCTTAAGATCGGAAGAGCGGUTCAGCAGGAAUGCCGAGACCGAUCTCGTATGCCGUCTTCTGCTTGAATGAUACGGCGACCACCGAGAUCTACACTCTUTCCCTACACGACGCUCTTCCGATCTAATACAGATCATGGCACGAG - 3'

Vector oligonucleotide:

5' - AGATCGGAAGAGCGTCGTGTAGGGAAAGAGTGTAGATCTCGGTGGTCGCCGTATCATTCAAGCAGAAGACGGCATACGAGATCGGTCTCGGCATTCCTGCTGAACCGCTCTTCCGATCT - 3'

Circularization reaction combines three DNA components, genomic circularization oligonucleotide, vector and targeted genomic DNA fragment:

```
3' - GAGCACGGUACTAGACATAATCTAGCCTTCUCGCAGCACATCCCTUTCTCACATCUAGAGCCACCAGCGGCAUAGTAAGTTCGTCTTCUGCCGTATGCTCUAGCCAGAGCCGUAAGGACGACTUGGCGAGAAGGCUAGAATTCUCCAACCGAAGGATGA - 5'

     CTCGTGCCATGATCTGTATT - 3'                                                                                                  5' -  TAAGAGGTTGGCTTCCTACT
     n                                                                                                                                                  n
     n               5'- AGATCGGAAGAGCGTCGTGTAGGGAAAGAGTGTAGATCTCGGTGGTCGCCGTATCATTCAAGCAGAAGACGGCATACGAGATCGGTCTCGGCATTCCTGCTGAACCGCTCTTCCGATCT - 3'             n
     n                                                                                                                                                  n
     nnnnnnnnnnnnnnnnnnnnnnnnnnnnnnnnnnnnnnnnnnnnnnnnnnnnnnnnnnnnnnnnnnnnnnnnnnnnnnnnnnnnnnnnnnnnnnnnnnnnnnnnnnnnnnnnnnnnnnnnnnnnnnnnnnnnnnnnnnnnnnnnnnnnnnnnnnn
```

(Only 20-mer target sites are specified in the genomic DNA fragment at nucleotide level. Sequence between target sites are depicted as generic nucleotide 'n'. Lower case represents minus-strand and HIHGER case represents plus-strand.)

1.3.2. Ligation

Thermostable ligase is used to ligate vector oligonucleotide to genomic DNA:

```
3' - GAGCACGGUACTAGACATAATCTAGCCTTCUCGCAGCACATCCCTUTCTCACATCUAGAGCCACCAGCGGCAUAGTAAGTTCGTCTTCUGCCGTATGCTCUAGCCAGAGCCGUAAGGACGACTUGGCGAGAAGGCUAGAATTCUCCAACCGAAGGATGA - 5'
     CTCGTGCCATGATCTGTATTAGATCGGAAGAGCGTCGTGTAGGGAAAGAGTGTAGATCTCGGTGGTCGCCGTATCATTCAAGCAGAAGACGGCATACGAGATCGGTCTCGGCATTCCTGCTGAACCGCTCTTCCGATCTTAAGAGGTTGGCTTCCTACT
     n                                                                                                                                                  n
     n                                                                                                                                                  n
     n                                                                                                                                                  n
     nnnnnnnnnnnnnnnnnnnnnnnnnnnnnnnnnnnnnnnnnnnnnnnnnnnnnnnnnnnnnnnnnnnnnnnnnnnnnnnnnnnnnnnnnnnnnnnnnnnnnnnnnnnnnnnnnnnnnnnnnnnnnnnnnnnnnnnnnnnnnnnnnnnnnnnnnnn
```

1.3.3. Denature

After heat denature, recombinant circle and genomic circularization oligonucleotide

```
3' - GAGCACGGUACTAGACATAATCTAGCCTTCUCGCAGCACATCCCTUTCTCACATCUAGAGCCACCAGCGGCAUAGTAAGTTCGTCTTCUGCCGTATGCTCUAGCCAGAGCCGUAAGGACGACTUGGCGAGAAGGCUAGAATTCUCCAACCGAAGGATGA - 5'

     CTCGTGCCATGATCTGTATTAGATCGGAAGAGCGTCGTGTAGGGAAAGAGTGTAGATCTCGGTGGTCGCCGTATCATTCAAGCAGAAGACGGCATACGAGATCGGTCTCGGCATTCCTGCTGAACCGCTCTTCCGATCTTAAGAGGTTGGCTTCCTACT
     n                                                                                                                                                  n
     n                                                                                                                                                  n
     n                                                                                                                                                  n
     nnnnnnnnnnnnnnnnnnnnnnnnnnnnnnnnnnnnnnnnnnnnnnnnnnnnnnnnnnnnnnnnnnnnnnnnnnnnnnnnnnnnnnnnnnnnnnnnnnnnnnnnnnnnnnnnnnnnnnnnnnnnnnnnnnnnnnnnnnnnnnnnnnnnnnnnnnnn
```

1.4. UDG

After Uracil-Excision reaction Uracils are roomed from the genomic circularization oligonucleotide. Oligo is fragmented to <20 nucleotides fragments:

```
3' - GAGCACGG ACTAGACATAATCTAGCCTTC CGCAGCACATCCCT TCTCACATC AGAGCCACCAGCGGCA AGTAAGTTCGTCTTC GCCGTATGCTC AGCCAGAGCCG AAGGACGACT GGCGAGAAGGC AGAATTC CCAACCGAAGGATGA - 5'

     CTCGTGCCATGATCTGTATTAGATCGGAAGAGCGTCGTGTAGGGAAAGAGTGTAGATCTCGGTGGTCGCCGTATCATTCAAGCAGAAGACGGCATACGAGATCGGTCTCGGCATTCCTGCTGAACCGCTCTTCCGATCTTAAGAGGTTGGCTTCCTACT
     n                                                                                                                                                  n
     n                                                                                                                                                  n
     n                                                                                                                                                  n
     nnnnnnnnnnnnnnnnnnnnnnnnnnnnnnnnnnnnnnnnnnnnnnnnnnnnnnnnnnnnnnnnnnnnnnnnnnnnnnnnnnnnnnnnnnnnnnnnnnnnnnnnnnnnnnnnnnnnnnnnnnnnnnnnnnnnnnnnnnnnnnnnnnnnnnnnnnn
```

1.5. Purification

Small oligo-fragments are removed using column purification and linear DNA is purified using exonucleose treatment leaving circular DNA intact:

```
     CTCGTGCCATGATCTGTATTAGATCGGAAGAGCGTCGTGTAGGGAAAGAGTGTAGATCTCGGTGGTCGCCGTATCATTCAAGCAGAAGACGGCATACGAGATCGGTCTCGGCATTCCTGCTGAACCGCTCTTCCGATCTTAAGAGGTTGGCTTCCTACT
     n                                                                                                                                                  n
     n                                                                                                                                                  n
     n                                                                                                                                                  n
     nnnnnnnnnnnnnnnnnnnnnnnnnnnnnnnnnnnnnnnnnnnnnnnnnnnnnnnnnnnnnnnnnnnnnnnnnnnnnnnnnnnnnnnnnnnnnnnnnnnnnnnnnnnnnnnnnnnnnnnnnnnnnnnnnnnnnnnnnnnnnnnnnnnnnnnnnnn
```

1.5. PCR

An optional PCR step can be applied to amplify the circular template:

Paired-end Enrichment Primer 1:
5' - AATGATACGGCGACCACCGAGATCTACACTCTTTCCCTACACGACGCTCTTCCGATCT - 3'

Paired-end Enrichment Primer 2:
5' - CAAGCAGAAGACGGCATACGAGATCGGTCTCGGCATTCCTGCTGAACCGCTCTTCCGATCT - 3'

PCR reaction:

```
5' -                                                                               CAAGCAGAAGACGGCATACGAGATCGGTCTCGGCATTCCTGCTGAACCGCTCTTCCGATCT - 3'
3' -                     TCTAGCCTTCTCGCAGCACATCCCTTTCTCACATCTAGAGCCACCAGCGGCATAGTAA - 5'
     CTCGTGCCATGATCTGTATTAGATCGGAAGAGCGTCGTGTAGGGAAAGAGTGTAGATCTCGGTGGTCGCCGTATCATTCAAGCAGAAGACGGCATACGAGATCGGTCTCGGCATTCCTGCTGAACCGCTCTTCCGATCTTAAGAGGTTGGCTTCCTACT
     n                                                                                                                                                             n
     n                                                                                                                                                             n
     n                                                                                                                                                             n
     nnnnnnnnnnnnnnnnnnnnnnnnnnnnnnnnnnnnnnnnnnnnnnnnnnnnnnnnnnnnnnnnnnnnnnnnnnnnnnnnnnnnnnnnnnnnnnnnnnnnnnnnnnnnnnnnnnnnnnnnnnnnnnnnnnnnnnnnnnnnnnnnnnnnnnnnnnnnnn
```

PCR product:

```
5' - CAAGCAGAAGACGGCATACGAGATCGGTCTCGGCATTCCTGCTGAACCGCTCTTCCGATCTTAAGAGGTTGGCTTCCTACTnnnnnnnCTCGTGCCATGATCTGTATTAGATCGGAAGAGCGTCGTGTAGGGAAAGAGTGTAGATCTCGGTGGTCGCCGTATCATT - 3'
3' - GTTCGTCTTCTGCCGTATGCTCTAGCCAGAGCCGTAAGGACGACTTGGCGAGAAGGCTAGAATTCTCCAACCGAAGGATGANNNNNNNGAGCACGGTACTAGACATAATCTAGCCTTCTCGCAGCACATCCCTTTCTCACATCTAGAGCCACCAGCGGCATAGTAA - 5'
```

2. CLUSTER GENERATION

2.0. Illumina sequencing oligonucleotides

Flow cell paired-end anchor oligonucleotides:

FC - AATGATACGGCGACCACCGAGAUCTACAC - 3'

3' - CACATCUAGAGCCACCAGCGGCATAGTAA - FC

‘C’: 5’ - PS-TTTTTTTTTTAATGATACGGCGACCACCGAGAUCTACAC - 3’ (U = 2-deoxyuridine)

FC - CAAGCAGAAGACGGCATACGAGAT - 3’

3' - TAGAGCATACGGCAGAAGACGAAC - FC

‘D’: 5’ - PS-TTTTTTTTTTCAAGCAGAAGACGGCATACGAGoxoAT - 3’, (Goxo = 8-oxoguanine)

Gemomic DNA Sequencing Primer for Read 1:

5' - ACACTCTTTCCCTACACGACGCTCTTCCGATCT - 3'

Multiplex

5' - ACACTCTTTCCCTACACGACGCTCTTCCGATC - 3'

Multiplex-T and Index

Same sequencing primer as generic illumina prep.

Gemomic DNA Sequencing Primer for Read 2:

5’ - CGGTCTCGGCATTCCTGCTGAACCGCTCTTCCGATCT - 3’

3' - TCTAGCCTTCTCGCCAAGTCGTCCTTACGGCTCTGGC - 5'

2.1. Bridge PCR

Circular templates are immobilized using regular Illumina solid phase amplification.

2.1.1. Anneal

Circular template is hybridized to Illumina Flow Cell oligo 'C':

```
                                                  3' - CACATCUAGAGCCACCAGCGGCATAGTAA - FC
      CTCGTGCCATGATCTGTATTAGATCGGAAGAGCGTCGTGTAGGGAAAGAGTGTAGATCTCGGTGGTCGCCGTATCATTCAAGCAGAAGACGGCATACGAGATCGGTCTCGGCATTCCTGCTGAACCGCTCTTCCGATCTTAAGAGGTTGGCTTCCTACT
      n                                                                                                                                                             n
      n                                                                                                                                                             n
      n                                                                                                                                                             n
      nnnnnnnnnnnnnnnnnnnnnnnnnnnnnnnnnnnnnnnnnnnnnnnnnnnnnnnnnnnnnnnnnnnnnnnnnnnnnnnnnnnnnnnnnnnnnnnnnnnnnnnnnnnnnnnnnnnnnnnnnnnnnnnnnnnnnnnnnnnnnnnnnnnnnnnnnnnnnn
```

2.1.3. Synthesize

DNA polymerase synthesizes the

```
                                                                                 FC  3'
                                                                                   ||
      GAGCACGGTACTAGACATAATCTAGCCTTCTCGCAGCACATCCCTTTCTCACATCUAGAGCCACCAGCGGCATAGTAAGTTCGTCTTCUGCCGTATGCTCTAGCCAGAGCCGUAAGGACGACTTGGCGAGAAGGCUAGAATTCTCCAACCGAAGGATGA
      CTCGTGCCATGATCTGTATTAGATCGGAAGAGCGTCGTGTAGGGAAAGAGTGTAGATCTCGGTGGTCGCCGTATCATTCAAGCAGAAGACGGCATACGAGATCGGTCTCGGCATTCCTGCTGAACCGCTCTTCCGATCTTAAGAGGTTGGCTTCCTACT
     Nn                                                                                                                                                             nN
     Nn                                                                                                                                                             nN
     Nn                                                                                                                                                             nN
     Nnnnnnnnnnnnnnnnnnnnnnnnnnnnnnnnnnnnnnnnnnnnnnnnnnnnnnnnnnnnnnnnnnnnnnnnnnnnnnnnnnnnnnnnnnnnnnnnnnnnnnnnnnnnnnnnnnnnnnnnnnnnnnnnnnnnnnnnnnnnnnnnnnnnnnnnnnnnnnnn
       NNNNNNNNNNNNNNNNNNNNNNNNNNNNNNNNNNNNNNNNNNNNNNNNNNNNNNNNNNNNNNNNNNNNNNNNNNNNNNNNNNNNNNNNNNNNNNNNNNNNNNNNNNNNNNNNNNNNNNNNNNNNNNNNNNNNNNNNNNNNNNNNNNNNNNNNNNNNN
```

2.1.4. Denature

After immobilization, template is denatured:

```
FC -  AATGATACGGCGACCACCGAGAUCTACACTCTTTCCCTACACGACGCTCTTCCGATCTAATACAGATCATGGCACGAGNNNNNNNNNNNNAGTAGGAAGCCAACCTCTTAAGATCGGAAGAGCGGTTCAGCAGGAATGCCGAGACCGATCTCGTATGCCGTCTTCTGCTTG - 3'
```

2.1.5. Bridge

Immobilized template forms a bridge to flow cell oligo 'D':

```
FC -  AATGATACGGCGACCACCGAGAUCTACACTCTTTCCCTACACGACGCTCTTCCGATCTAATACAGATCATGGCACGAGNNNNNNNNNNNNAGTAGGAAGCCAACCTCTTAAGATCGGAAGAGCGGTTCAGCAGGAATGCCGAGACCGATCTCGTATGCCGTCTTCTGCTTG - 3'
3' -                                                                                                                                                               TAGAGCATACGGCAGAAGACGAAC - FC
```

2.1.6. Synthesize

Synthesis of the second strand takes place in the flow cell:

```
FC -  AATGATACGGCGACCACCGAGAUCTACACTCTTTCCCTACACGACGCTCTTCCGATCTAATACAGATCATGGCACGAGNNNNNNNNNNNAGTAGGAAGCCAACCTCTTAAGATCGGAAGAGCGGTTCAGCAGGAATGCCGAGACCGATCTCGTATGCCGTCTTCTGCTTG - 3'
3' -  TTACTATGCCGCTGGTGGCTCTAGATGTGAGAAAGGGATGTGCTGCGAGAAGGCTAGATTATGTCTAGTACCGTGCTCnnnnnnnnnnnTCATCCTTCGGTTGGAGAATTCTAGCCTTCTCGCCAAGTCGTCCTTACGGCTCTGGCTAGAGCATACGGCAGAAGACGAAC - FC
```

2.1.7. Denature

Immobilized fragments are released by denaturing:

```
FC -  AATGATACGGCGACCACCGAGAUCTACACTCTTTCCCTACACGACGCTCTTCCGATCTAATACAGATCATGGCACGAGNNNNNNNNNNNAGTAGGAAGCCAACCTCTTAAGATCGGAAGAGCGGTTCAGCAGGAATGCCGAGACCGATCTCGTATGCCGTCTTCTGCTTG - 3'

3' -  TTACTATGCCGCTGGTGGCTCTAGATGTGAGAAAGGGATGTGCTGCGAGAAGGCTAGATTATGTCTAGTACCGTGCTCnnnnnnnnnnnTCATCCTTCGGTTGGAGAATTCTAGCCTTCTCGCCAAGTCGTCCTTACGGCTCTGGCTAGAGCATACGGCAGAAGACGAAC - FC
```

3. SEQUENCING

<u>3.1. Anneal sequencing primer Read 1</u>

Illumina paired end sequencing primer 1 is hybridized to clustered DNA:

```
5' -                             ACACTCTTTCCCTACACGACGCTCTTCCGATCT                                                                                                                         - 3'
3' -  TTACTATGCCGCTGGTGGCTCTAGATGTGAGAAAGGGATGTGCTGCGAGAAGGCTAGATTATGTCTAGTACCGTGCTCnnnnnnnnnnnTCATCCTTCGGTTGGAGAATTCTAGCCTTCTCGCCAAGTCGTCCTTACGGCTCTGGCTAGAGCATACGGCAGAAGACGAAC - FC
```

<u>3.2. Cyclic sequencing-by-synthesis Read 1</u>

Sequencing-by-synthesis cycles are performed using standard Illumina protocol to interrogate the DNA sequence of the template:

```
5' -                             ACACTCTTTCCCTACACGACGCTCTTCCGATCT - - - - - - - - >                                                                                                       - 3'
3' -  TTACTATGCCGCTGGTGGCTCTAGATGTGAGAAAGGGATGTGCTGCGAGAAGGCTAGATTATGTCTAGTACCGTGCTCnnnnnnnnnnnTCATCCTTCGGTTGGAGAATTCTAGCCTTCTCGCCAAGTCGTCCTTACGGCTCTGGCTAGAGCATACGGCAGAAGACGAAC - FC
```

(First 20 bases of the sequence represent genomic DNA site of the target.)

<u>3.3. Anneal sequencing primer Read 2</u>

Illumina paired end sequencing primer 2 is hybridized to clustered DNA:

```
FC -  AATGATACGGCGACCACCGAGAUCTACACTCTTTCCCTACACGACGCTCTTCCGATCTAATACAGATCATGGCACGAGNNNNNNNNNNNAGTAGGAAGCCAACCTCTTAAGATCGGAAGAGCGGTTCAGCAGGAATGCCGAGACCGATCTCGTATGCCGTCTTCTGCTTG - 3'
3' -                                                                                                              TCTAGCCTTCTCGCCAAGTCGTCCTTACGGCTCTGGC                         - 5'
```

<u>3.4. Cyclic sequencing-by-synthesis Read 2</u>

Sequencing-by-synthesis cycles are performed using standard Illumina protocol to interrogate the DNA sequence of the template:

```
FC -  AATGATACGGCGACCACCGAGAUCTACACTCTTTCCCTACACGACGCTCTTCCGATCTAATACAGATCATGGCACGAGNNNNNNNNNNNAGTAGGAAGCCAACCTCTTAAGATCGGAAGAGCGGTTCAGCAGGAATGCCGAGACCGATCTCGTATGCCGTCTTCTGCTTG - 3'
3' -                                                                                                < - - - - - - TCTAGCCTTCTCGCCAAGTCGTCCTTACGGCTCTGGC                         - 5'
```

(First 20 bases of the sequence represent genomic DNA site of the target.)
